# Evaluation of hematopoietic cells and myeloid/erythroid ratio in the bone marrow of the pheasant *(Phasianus colchicus)*

**Published:** 2013

**Authors:** Mina Tadjalli, Saeed Nazifi, Rahil Haghjoo

**Affiliations:** 1 *Department of Basic Sciences, School of Veterinary Medicine, Shiraz University, Shiraz, Iran; *; 2 *Department of Clinical Studies, School of Veterinary Medicine, Shiraz University, Shiraz, Iran.*

**Keywords:** Bone marrow, Hematopoietic cells morphology, Myeloid/erythroid ratio, Pheasant (*Phasianus colchicus*)

## Abstract

In order to study the normal hematopoiesis, cellular components and myeloid/erythroid (M/E) ratio in the bone marrow of the pheasant *(Phasianus colchicus)*, bone marrow samples were collected from the proximal tibiotarsus bone of 16 clinically healthy adult pheasant. The bone marrow smears were stained using the Giemsa stain. The results indicated that the development and formation of blood cells in the bone marrow of pheasant were similar to other birds, whereas the morphology of the cells was similar to chickens, ducks, quail, and black-head gull. The mean M/E ratio was 1.24, the mean erythroid percentage was 42.24, the mean myeloid percentage was 52.62, and the mean percentage of all other cells percentage was 5.38. There was no significant difference in any of the cellular composition between male and female.

## Introduction

Bone marrow examination can provide valuable information about hematopoietic status.^1^ The usefulness of bone marrow aspiration as a diagnostic tool depends on proper collection and handling of the sample and on knowledge of normal marrow morphology.^2^ It is imperative that a blood sample is always collected together with the bone marrow specimen for proper comparative evaluation.^1^ Various indicators of marrow examination in avian patients include: nonregenerative anemias, thrombocytopenias, heteropenias, pancytopenias, suspected leukaemia and other unexplained cellular changes in the peripheral blood.^3^ Fine structure and hematopoietic cell morphology of bone marrow of chickens, pigeon, ducks, Japanese quails and black head gull have been investigated by Campbell,^3^^,^^4^ Campbell and Coles,^5^ Tadjalli *et al*.,^6^ Nazifi *et al*.,^7^ and Tadjalli *et al*.^8^ Hematology and blood chemistry of gulls were reported by Averbeck^9^ and Work,^10^ but there is no information about the hematopoietic cells of the pheasant. The purpose of the present study, therefore, was to determine the bone marrow cell morphology and M/E ratio in pheasant.

## Materials and Methods

Bone marrow aspirations were obtained from 16 clinically healthy adult pheasant (8 male and 8 female). All birds were free of hematological abnormalities on peripheral blood examination. The medial aspect of the proximal tibiotarsus bone, just below the femoral-tibiotarsal joint, was aseptically prepared and 22 gauge disposable marrow aspiration needle were used to obtain samples. The area was anaesthetized locally by subcutaneous infiltration of 1-1.5 mL of 2% lignocaine HCl (Aburaihan pharmaceutical Co., Tehran, Iran) over the periosteum. The aspiration biopsy needle was held perpendicular to the bone and advanced in to the marrow space by applying light pressure and using slight rotatory motions. With the needle in the marrow space, the stylet was removed and a syringe was locked into the needle. The samples were collected into 5 mL syringes containing EDTA. At least five air-dried wedge slides of bone marrow smears were prepared from each pheasant. Slides were stained with Giemsa and were evaluated for cellularity and classification of erythroid, myeloid and thrombocytic precursors. Each sample was used for a 500-cell differential count to classify the marrow precursors in each cell series and to determine myeloid: erythroid (M/E) ratios for each pheasant. The M/E ratio was determined by dividing the total of all the nucleated cells of the granulocytic series by the total of all the nucleated cells of the erythrocytic series.^11^ The classification of the erythroid series included rubriblasts, prorubricytes, basophilic rubriccytes, early polychromatophilic rubricytes and late polychromatophilic rubricytes. The classification of the myeloid series included myeloblasts, promyelocytes, metamyelocytes, bands and segmenters. The results were expressed as mean ± SEM. All data were processed using SPSS (Version 16.0 for windows, SPSS Inc., Chicago, IL, USA). The results were analyzed using *t*-test for comparison between two sexes. Statistical significance was set at *p* < 0.05.

## Results

The cellular composition of the bone marrow of pheasant is presented in Table 1. The mean value for the M/E ratio was 1.24.

**Fig. 1:**
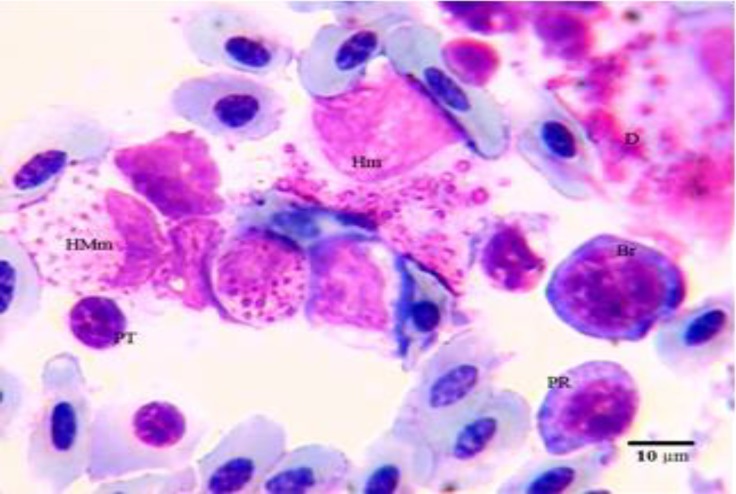
Photomicrograph of hematopoietic cells in bone marrow of male pheasant. Giemsa staining. PR: early polychromatic rubricyte; Br: basophilic rubricyte; Hm: heterophilic myelocyte; HMm: heterophilic metamyelocyte; D: degenerated cell; PT: prothrombocyte.

**Fig. 2:**
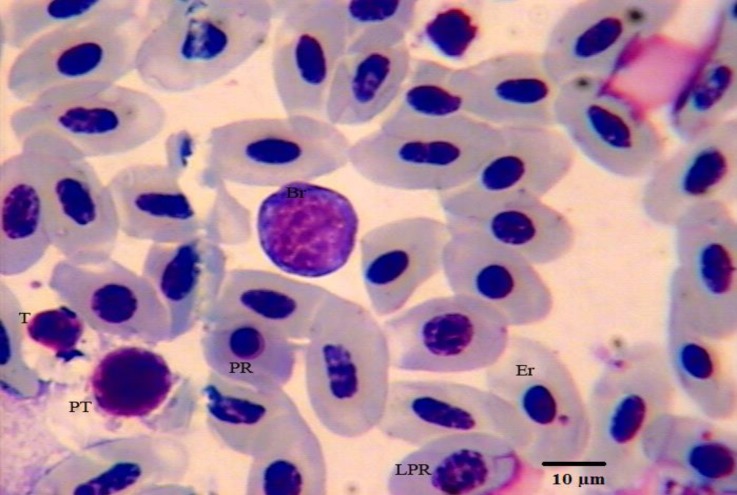
Photomicrograph of hematopoietic cells in bone marrow of female pheasant. Giemsa staining. LPR: late polychromatophilic rubricyte; PR: early polychromatic rubricyte; Br: basophilic rubricyte; Er: erythrocyte; PT: prothrombocyte; T: thrombocyte.

The mean percentage for erythroid and myeloid cells were 42.24 and 52.62, respectively. The finding of this study revealed that the highest percentage of cells were early polychromatophilic rubricytes in the erythroid series and myelocytes in the myeloid series. Rubriblasts were big cells with large central round nuclei with nucleoli. The nucleus-cytoplasm ratio was high. The cytoplasm was deeply basophilic and vacuolated. Pro-rubricytes resembled rubriblasts, but their chromatin was more dense, nucleoli were indistinct and their cytoplasm very deeply basophilic. Basophilic rubricytes were smaller than prorubricytes with a round nucleus containing clumped chromatin (Figs. 1 and 2). Early poly-chromatophilic rubricytes were round cells with a grey (basophilic to slightly eosinophilic) cytoplasm. The nucleus of these cells was small in relation to the cytoplasm and had clumped chromatin (Figs. 1 and 2). Late poly-chromatophilic rubricytes were approximately oval in shape with a nucleus round to slightly oval containing irregularly clumped chromatin (Fig. 2).

**Fig. 3:**
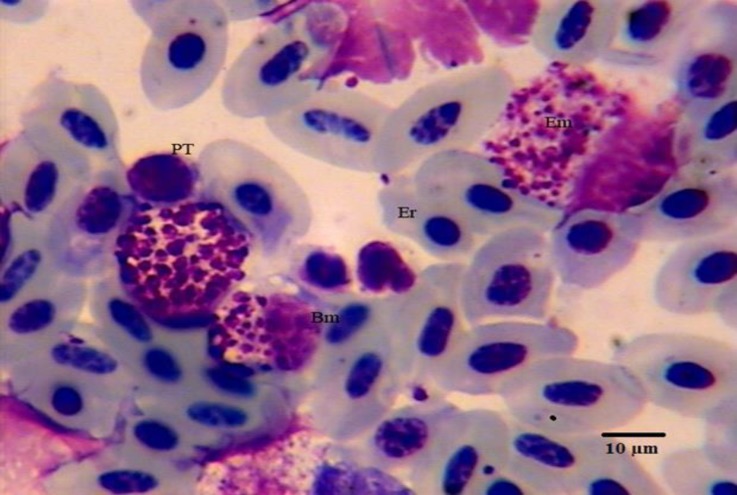
Photomicrograph of hematopoietic cells in bone marrow of male pheasant. Giemsa staining. Er: erythrocyte; Em: eosinophilic myelocyte; Bm: basophilic myelocyte; PT: prothrombocyte.

Myeloblasts were large and round with a narrow rim of blue cytoplasm. Their nucleus was round with a reticular chromatin and prominent nucleoli. Promyelocytes were large round cells with light blue cytoplasm and eccentric round nucleus. Their cytoplasm contained dark magenta granules. Myelocytes were smaller than promyelocytes. They had a spherical shape with an eccentric oval nucleus. Their cytoplasm contained secondary granules (specific granules) which could be classified as the heterophil (Fig. 1), eosinophil (Fig. 3) or basophilic (Fig. 3) series. Heterophilic, eosinophilic and basophilic myelocytes contained less than half the definitive number of mature granules. Eosinophilic myelocytes lacked the magenta granules (Fig. 3).

Metamyelocytes were smaller than their precursor cells. Their nucleus was slightly indented or bean shape and their cytoplasm had more than half the definitive number of specific granules (Fig. 1). Band cells resembled the mature granulocyte but lacked the lobed nucleus. 

**Table 1:** Cellular composition of the bone marrow of female (n=8) and male (n=8) pheasant.

**Cells (%)**	**Female**	**Male**	**Cells (%)**	**Female**	**Male**	**Cells (%)**	**Female**	**Male**
Rubriblast	1.88 ± 0.26	2.42 ± 0.42	Myeloblast	0.66 ± 0.16	0.50 ± 0.18	Mitotic cell	0.77 ±0. 22	0.57 ± 0.20
Prorubricyte	6.89 ± 0.42	7.00 ± 0.61	Promyelocyte	4.11 ± 0.35	3.86 ± 0.34	Osteoclast	0.66 ± 0.23	0.71 ± 0.28
Basophilic rubricyte	5.44 ± 0.44	4.57 ±0. 57	Myelocyte, basophilic	6.78 ± 0.22	7.70 ± 0.27	Plasma cell	0.66 ± 0.16	0.71 ± 0.28
Early polychromatophilic rubricyte	22.11 ± 0.51	21.71 ± 0.68	Myelocyte	27.22 ± 0.32	27.57 ± 0.71	Thrombocyte	1.44 ± 0.19	1.42 ± 0.36
Late polychromatophilic rubricyte	6.33 ± 0.23	6.14 ± 0.45	Metamyelocyte	6.44 ± 0.37	6.57 ± 0.57	Monocyte	0.44 ± 0.17	1.00 ± 0.21
Total erythroid cells	42.65 ± 0.37	41.84 ± 0.54	Band cell	6.00 ± 0.28	5.14 ±0. 26	Lymphocyte	0.55 ± 0.18	0.71 ± 0.28
			Heterophil	0.44 ± 0.17	1.00 ± 0.30	Degenerate cell	0.56 ± 0.17	0.57 ± 0. 20
			Eosinophil	0.55 ± 0.17	0.57 ± 0.20	Total other cells	5.08 ± 0.19	5.69 ± 0.26
			Basophil	-	0.14 ± 0.14	M/E ratio	1.24 ± 0.36	-
			Total myeloid cells	52.20 ± 0.23	53.05 ± 0.33			

**Fig. 4:**
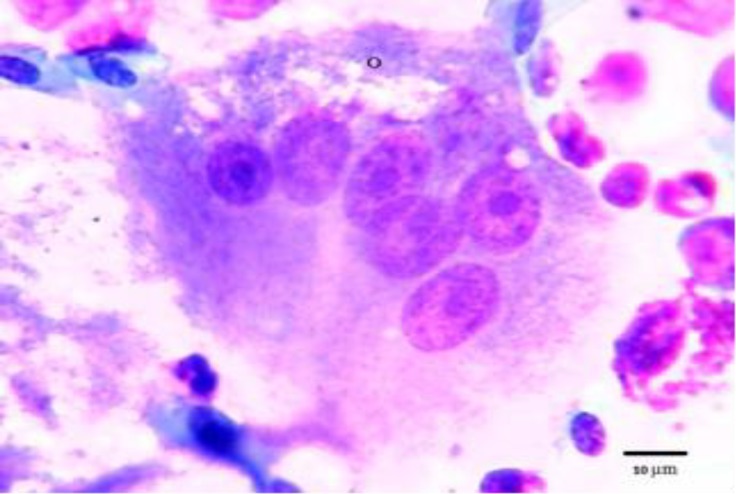
Photomicrograph of osteoclast (o) in bone marrow of male pheasant. Giemsa staining.

Thromboblasts were not observed in pheasant bone marrow, but, prothrombocytes and thrombocytes were seen in low percentages (Figs. 1, 2 and 3). Promonocytes were large cells with clear blue cytoplasm containing granules and round nuclei with a reticular nuclear chromatin. The lymphoblast and prolymphocyte were not observed in pheasant bone marrow samples. Plasma cells were round to oval cells with a round, eccentrically placed nucleus. A pale area of cytoplasm was observed near one side of the nucleus. Osteoclasts were large multinucleated giant amoeboid cells. Their cytoplasm consisted of eosinophilic granules in different shapes and size, and was also vacuolated. Their nuclei were round to oval with finely granular chromatin and prominent nucleoli (Fig.4). There was no significant difference in any of the cellular composition between male and female.

## Discussion

A unique feature of avian species is that erythropoiesis and possibly thrombopoiesis occur within vascular sinuses, but granulopoiesis take place outside the vascular sinuses.^12^

The development and formation of blood cells in the bone marrow of pheasant were similar to other birds,^3^^,^^5^ whereas the morphology of the cells was similar to chickens, pigeons, ducks, quails and black headed gull.^4^^,^^6^^-^^8^ The rubriblasts in pheasant bone marrows were similar to hemocytoblasts in chickens, pigeons, ducks, quails and black head gull.^4^^-^^8^ Campbell and Coles indicated that the stem cell for erythrocytes was the rubriblast (erythroblast).^5^ In the bone marrow of pheasant, prorubricytes resembled rubriblasts, but the nucleolus was indistinct or absent. These prorubricytes were similar to those in chickens, ducks, quails and black head gull.^5^^-^^8^ In the bone marrow of pheasant, basophilic rubricytes, early polychromatophilic rubricytes and late polychromatophilic rubricytes were similar to the respective cells in chicken, pigeon, ducks, adult quail and black head gull.^4^^,^^6^^-^^8^ Comparatively, cellular elements of erythropoiesis in birds are similar to those of mammals.^1^^,^^3^^,^^5^ The findings of this study revealed that the highest percentages of cells were early polychromatophilic rubricytes and the lowest percentage were rubriblasts in the erythroid series. These findings were similar to those of Campbell and Coles in chickens,^5^ Tadjalli *et al*. in ducks,^6^ Nazifi *et al*. in quails^7^ and Tadjalli *et al*. in black head gull.^8^

The pheasant’s granulocytic series was similar to those of other birds and mammals^1^^,^^5^ and in particular to chickens,^3^^-^^5^ ducks,^6^ quails^7^ and black head gull.^8^ It is believed that promyelocytes and pro-granulocytes were identical.^3^^,^^5^ In the present study, the highest percentages of cells in the myeloid series were related to myelocytes a finding similar to that of Tadjalli *et al*. in ducks.^6^

Similar to ducks,^6^ quails^7^ and black head gull^8^ monoblasts were not observed in the bone marrow of pheasant, but promonocytes were seen in low percentages. Indeed, monoblasts have not been recognized in avian bone marrow.^12^ It is likely that precursors of monocytes and heterophils are similar in the early stages, as in mammals, and cannot be distinguished by light microscopy^3^^,^^12^ Lymphocytes were observed in bone marrow of pheasant similar to those in quails^7^ and black head gull.^8^ Campbell reported that bone marrow of adult chickens and pigeons contained numerous accumulations of lymphatic tissues.^4^ By contrast, Bounous and Stedman reported that in adult birds, blood lymphocytes probably arise mostly from peripheral lymphoid tissues including the spleen, caecal tonsils and other gut-associated lymphoid tissue.^12^ The incidence of promonocytes, mitotic cells, plasma cells and osteoclasts were comparable to other domestic species.^1^

Unlike mammalian platelets, which are cytoplasmic fragments of megakaryocytes, avian thrombocytes are derived from mononuclear precursor cells.^5^^,^^12^

The mean value for M/E ratio in the bone marrow of pheasant was 1.24, which was comparable to that in duck (1.00),^6^ black head gull (1.23),^8^ camel (1.21),^13^ and dog (0.75-2.50), cat (1.20-2.20), horse (0.50-1.50), cattle (0.31-1.85) and sheep (0.77-1.68).^1^ However it differs from quail (0.37),^7^ and goat (0.69).^1^ The cellularity of the bone marrow smears was comparable to that of other domestic species.^1^^,^^7^^,^^13^^-^^15^
